# Identifying the ligated amino acid of archaeal tRNAs based on positions outside the anticodon

**DOI:** 10.1261/rna.053777.115

**Published:** 2016-10

**Authors:** Tal Galili, Hila Gingold, Shaul Shaul, Yoav Benjamini

**Affiliations:** 1 Department of Statistics and Operations Research, The Sackler Faculty of Exact Sciences, Tel Aviv University, Tel Aviv, Ramat-Aviv 69978, Israel; 2 Department of Molecular Genetics, Weizmann Institute of Science, Rehovot 76100, Israel; 3 The Edmond J. Safra Center for Bioinformatics and The Sagol School for Neuroscience, Tel Aviv University, Ramat-Aviv 69978, Israel

**Keywords:** RNA code, second genetic code, evolution, discriminate positions

## Abstract

Proper recognition of tRNAs by their aminoacyl-tRNA synthetase is essential for translation accuracy. Following evidence that the enzymes can recognize the correct tRNA even when anticodon information is masked, we search for additional nucleotide positions within the tRNA molecule that potentially contain information for amino acid identification. Analyzing 3936 sequences of tRNA genes from 86 archaeal species, we show that the tRNAs’ cognate amino acids can be identified by the information embedded in the tRNAs’ nucleotide positions without relying on the anticodon information. We present a small set of six to 10 informative positions along the tRNA, which allow for amino acid identification accuracy of 90.6% to 97.4%, respectively. We inspected tRNAs for each of the 20 amino acid types for such informative positions and found that tRNA genes for some amino acids are distinguishable from others by as few as one or two positions. The informative nucleotide positions are in agreement with nucleotide positions that were experimentally shown to affect the loaded amino acid identity. Interestingly, the knowledge gained from the tRNA genes of one archaeal phylum does not extrapolate well to another phylum. Furthermore, each species has a unique ensemble of nucleotides in the informative tRNA positions, and the similarity between the sets of positions of two distinct species reflects their evolutionary distance. Hence, we term this set of informative positions a “tRNA cipher.” It is tempting to suggest that the diverging code identified here might also serve the aminoacyl tRNA synthetase in the task of tRNA recognition.

## INTRODUCTION

The correct incorporation of an amino acid into a newly synthesized peptide requires both correct pairing between a cognate tRNA to the codon in the open A-site of the ribosome and correct charging of the tRNA with the amino acid that corresponds to its anticodon triplet. This process is characterized by a low error rate of 10^−4^–10^−5^ (Ibba and Söll 2000; Zaher and Green 2009). The selective charging of an amino acid onto the proper tRNA is performed by aminoacyl-tRNA synthetase enzymes (aaRS), relying on information stored inside and outside the anticodon (Francklyn and Schimmel 1990; McClain 1993; Martinis and Schimmel 1995; Giegé et al. 1998). Earlier studies revealed that even a single base pair substitution in the acceptor helix of tRNAs can manipulate the specificity of aminoacylation (Hou and Schimmel 1988; Frugier et al. 1992). Position N73, which precedes the CCA sequences at the 3′ end of the tRNAs, was shown to mediate the recognition of tRNAs by their cognate aminoacyl-tRNA synthetase, both computationally and experimentally. For instance, the discriminator base N73 of tRNA^His^ was found to be occupied with cytidine in all the Archaebacteria and Eubacteria and was further shown in vitro and in vivo to play a major role in the recognition between tRNA^His^ and its cognate histidyl-tRNA synthetase (Yan and Francklyn 1994). It is therefore well known as the “discriminator base” position. In addition to such identity elements, which allow tRNA-aaRS recognition, other nucleotide positions were suggested to constitute “negative identity elements” that serve in preventing the recognition of a given tRNA by the 19 noncognate aaRS types (Pak et al. 1994; Jako et al. 2007). The part of the tRNA outside the anticodon, determining its specificity to the aaRS, was termed by Schimmel et al. (1993) the generalized “operational RNA code” for amino acids and even “the second genetic code” (De Duve 1988; Schimmel et al. 1993).

The operational RNA code was hypothesized to serve as an aminoacylation barrier across different species and different phyla (Ribas de Pouplana and Schimmel 2001), emphasizing its species-specific nature. In particular, it is expected that the positions and nucleotides used for recognition of tRNAs by their cognate aminoacyl-tRNA synthetases (“determinants”) may vary between distant kingdoms (e.g., species from Archaea vs. Bacteria). Indeed, it has been experimentally shown that a tRNA synthetase from one domain of life will often, but not always (Xu et al. 2001), fail to aminoacylate tRNAs from other domains (Ripmaster et al. 1995; Kobayashi et al. 2003; Namgoong et al. 2007). Other studies showed that the aminoacylation specificity of some tRNA types, among distant species, may be determined by the very same base pair in their acceptor stem. This was demonstrated by both in vivo and in vitro (Giegé et al. 1998), as well as computational analysis (Shaul et al. 2010; Szenes and Pál 2012). Altogether, these studies suggest that the operational RNA code is common across species in some cases and species-specific in others. As a result, positions and/or nucleotides on the tRNAs that were experimentally shown to affect aminoacylation specificity in one or more species may, or may not, extrapolate to newly examined species.

While tRNA molecules traditionally have been discussed in the context of the translation process, it is now clear that they play additional major roles in living cells. For instance, tRNA molecules are involved in cell signaling pathways and stress response programs (Phizicky and Hopper 2010; Raina and Ibba 2014), and in regulation of apoptosis (Mei et al. 2010). Furthermore, the advance in high-throughput sequencing technologies has led to the identification of tRNA-derived RNA fragments in many species. Such a tRNA-derived RNA fragment, originating from tRNA^Val^, was shown to inhibit translation in the halophilic archaeon *Haloferax volcanii*, under environmental stress conditions, by interfering with peptidyl transferase activity (Gebetsberger et al. 2012). In eukaryotes, tRNA-derived RNA fragments are also implicated in important stages of cancer formation and progression, and in neurodegeneration (Karaca et al. 2014; Mleczko et al. 2014; Schaffer et al. 2014).

This work aims to explore the properties of the operational RNA code by employing statistical analysis of tRNA genes data. Specifically, we are looking for positions along the tRNAs that allow the prediction of their associated amino acid identity. The anticodon information was excluded from our analysis since it allows, by itself, a perfect prediction of the identity of the tRNA's corresponding amino acid, thus masking any additional potential positions. By excluding the anticodon from the analysis, our statistical modeling strategy enables us to detect potentially informative positions in other regions of the tRNA sequence.

We performed a statistical analysis on a comprehensive set of 3,936 tRNA gene sequences from 86 archaeal species (Chan and Lowe 2009) (see Supplemental Information S1,S2 for data preparation). We identified informative positions on the tRNA gene sequences, outside the anticodon, that contain enough information to discern (statistically) between tRNAs for one amino acid and tRNAs for all the other amino acids. We term the set of nucleotides residing in these informative positions as the “tRNA code.” The tRNA code allows for the discrimination between tRNAs for a given amino acid and all other tRNAs. Hence, it holds the needed information that (potentially) allows for the selective charging of tRNAs by their corresponding aaRS.

We find that a small number of informative positions on the tRNA genes contains enough information to give a good prediction of their ligated amino acid, and that tRNAs for distinct amino acids are characterized by various numbers of informative positions. Specifically, we reveal that tRNAs for four amino acids can be distinguished from tRNAs of other amino acids, using tRNA code embedded in only one (His, Tyr) or two (Gln, Leu) informative positions on the tRNA. Each such tRNA code is universally shared by all archaeal species in our data. In contrast, the informative positions in tRNAs for other amino acids do not appear to use the same informative positions and/or tRNA code across archaeal species. We compared our statistical predictions with available experimental evidences and found them to be consistent.

## RESULTS

### The cognate amino acid of tRNAs can be identified by a code embedded in a small set of informative (non-anticodon) nucleotide positions

We wish to find informative positions on archaeal tRNA genes, outside the anticodon, that contain information about the identity of their ligated amino acids. For this purpose, we construct statistical models that can predict the cognate amino acid, of a given tRNA, based on positions around the acceptor stem, D-domain and the TΨC-domain. The prediction models were created based on 3936 tRNA gene sequences, from a database of 86 archaeal species (Chan and Lowe 2009), using the CART method for classification decision-tree models (Breiman et al. 1984). A similar prediction model was previously created (Shaul et al. 2010) on a smaller set of 21 archaeal species based on the 15 nucleotide (nt) positions of the acceptor stem, achieving amino-acid identification accuracy of 89.3%. We replicated these findings on an extended data set of 86 archaeal species, achieving 88.87% identification accuracy (when using only the positions of the acceptor stem). The identification accuracy was measured as the percentage of tRNA sequences for which the model correctly identified their ligated amino acid. Note that in both studies, the same sequences were used both to construct the statistical prediction model and for its assessment, known as “within-sample” accuracy (see Supplemental Information S1, S2, S4).

To further improve the identification accuracy, we expand the set of 15 nt positions of the acceptor stem by adding 28 more nucleotide positions from the D-domain and the TΨC-domain, while excluding positions from the anticodon stem, the variable loop, and any highly conserved positions within these domains (see Supplemental Figs. S3–S5). A prediction model based on the full set of 43 aligned nucleotide positions, for all 3936 tRNA genes, reaches an identification accuracy of 99.92%.

**FIGURE 1.**
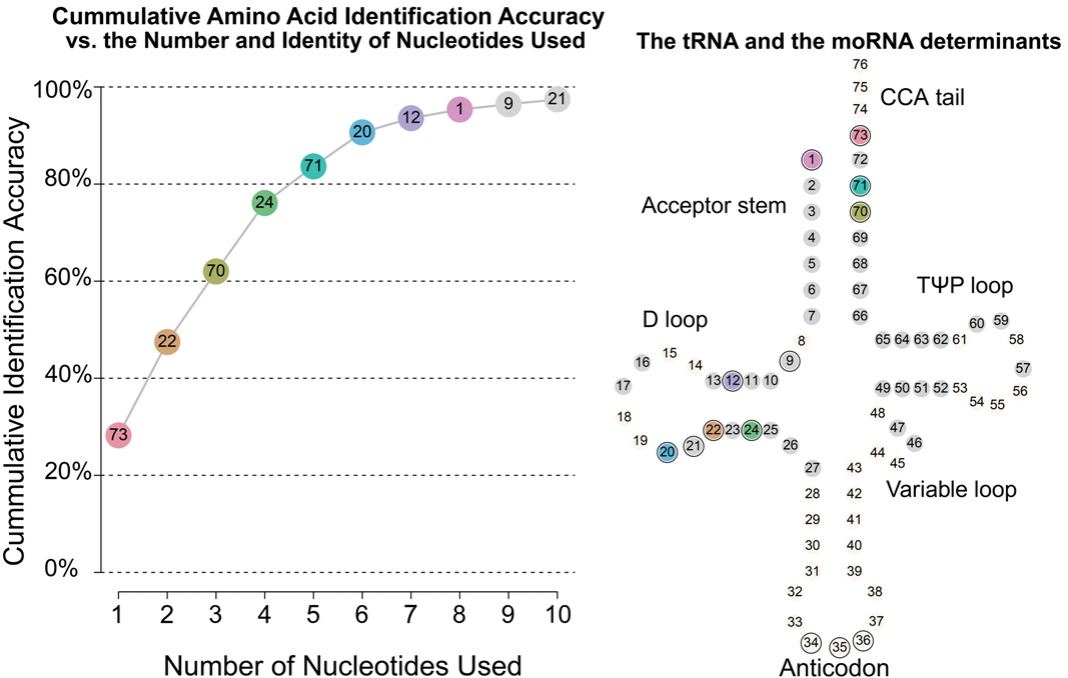
The cumulative identification accuracy (in %) of predicting the identity of the corresponding amino acids of tRNAs, based on nucleotide positions located outside the anticodon. The 43 considered nucleotide positions on the tRNA (*right* panel) are either grayed or colored. The Pareto plot (*left* panel) shows the incremental identification accuracy of the “most informative” positions (i.e., when sequentially adding these positions to the decision-tree prediction model).

While the set of 43 positions allows for substantial prediction accuracy, it is obvious that three nucleotide positions can be enough to identify the cognate amino acids of all the tRNAs (e.g., the anticodon). The existence of this known universal code motivated us to search (among the 43 positions) for a smaller subset of informative positions that might offer a high identification accuracy of the tRNAs’ ligated amino acids. To find a subset of informative positions that can predict the identity of the cognate amino acid of each tRNA gene, we search for a parsimonious prediction model using a greedy forward selection method. Accordingly, the most informative position among the available positions is chosen first, then the second most informative position is added to the model, and so on. This is done concurrently with tRNAs for all amino acids and from all species. The resulting model first chose base position N73, then added N22 as the best second position, and so on (see left panel of Fig. 1 for the full list of positions). We note that N73 is known as the “discriminator position,” for its important role in the recognition between tRNAs and their cognate aaRS (Crothers et al. 1972).

We find that a subset of ten nucleotide positions can be used to build a prediction model with a high (within sample) identification accuracy of 97.43%. By further restricting the size of the set of positions to 8, 6, and 3, we achieve an identification accuracy of 95.35%, 90.65%, and 61.99%, respectively (see Fig. 1).

Our statistical model is able to locate informative nucleotide positions on the tRNAs and, with them, predict for a given tRNA gene its cognate amino acid. The high identification accuracy, demonstrated in Figure 1, supports the hypothesis that positions outside the anticodon contain the needed information for matching a tRNA to its corresponding amino acid in the archaeal species. Hence, this combination of informative positions and their associated tRNA code offers a potential surrogate model for the “second genetic code” (Schimmel et al. 1993).

### tRNAs from distinct phyla use a different code in their informative positions

In the previous section, we found that a small set of informative positions, outside the anticodon, contain information that could potentially mirror the tRNA-aaRS recognition process, across all amino acids and archaeal species. We choose the set of eight informative nucleotide positions that yield amino acid identification accuracy (within sample) of 95.35%, and term it “the minimal operational RNA,” henceforth *moRNA* code. (See Fig. 1 for the positions of these nucleotides along the tRNA structure, and Supplemental Fig. S9 for the relative nucleotide frequencies in these eight positions.)

Next, we explore the ability of a chosen set of eight informative positions of one set of tRNA genes to predict the cognate amino acid identity of an unseen, separate set of tRNA genes. For that purpose, we randomly sample a set of 2/3 of the 3936 tRNA sequences and search for the most informative eight positions that can predict the cognate amino acid identity of tRNAs from this training set (using the same forward selection procedure as before). With these eight chosen positions from the training data, we construct a set of rules (using CART, see Materials and Methods) for predicting the tRNAs' cognate amino acid identity. We evaluated the identification accuracy of these rules on the remaining one-third of the tRNA (test-set) genes. This process is repeated 100 times, and the identification accuracy of predicting the identity of the cognate amino acid of tRNAs from the test-set was found to be 93%, on average.

This tRNA-cross-validation analysis (“t-CV,” see Materials and Methods) is naïve, as it ignores information regarding the species origin of each tRNA gene (i.e., ignoring a possible species-related dependency structure of the code in the tRNAs). We, therefore, repeat the analysis on tRNAs from a randomly chosen group of species of Archaea (s-CV, see Materials and Methods). We use 57 of 86 species for the training set in order to mimic the number of species in the largest represented phylum in our data. The prediction accuracy of this model, for the remaining species, yields a similarly high identification accuracy of 92.9% (on average over 100 such random samples).

The success rates of choosing eight positions using the full set of tRNA genes (95.3%) and the subset of tRNA genes in the cross-validations (∼93% for both t-CV and s-CV) are largely and significantly different from the success rates derived after permuting randomly the identity of the ligated amino acids (see Materials and Methods): 49.15% and 6.9%, respectively.

An intriguing question is whether the information needed for aaRS-tRNAs recognition is conserved between distinct phyla. Practically, we examine the performance of a model, developed on data from tRNA genes of one phylum, to predict the identity of the cognate amino acids of tRNA genes from another phylum (f-CV, see Materials and Methods).

Most of the species in our data (82 of 86 species) belong to one of the two phyla, either Euryarchaeota or Crenarchaeota. Building a model of moRNA code using 57 species of the Euryarchaeota phylum (2597 tRNAs) achieves an amino acid identification accuracy of only 74.1% for predicting the identity of the corresponding amino acid of the tRNAs in the remaining 29 archaeal species (outside the Euryarchaeota phylum). A similar model, using the 25 species from the Crenarchaeota phylum (1159 tRNAs), achieves an amino acid identification accuracy of only 52.8% on the tRNA genes of the remaining 61 archaeal species.

If the code embedded in the moRNA positions was universal across all species, as is the case for the genetic code embedded in the anticodon, then we would also get close to 100% identification accuracy of the tRNAs’ cognate amino acids identity across phyla (f-CV). For example, this would be the case if there were a copy of the anticodon in other positions on the tRNA molecule. In contrast, if the information in the positions was just pure noise, we would not get anywhere near the 93% prediction accuracy that was achieved in the t-CV; it would have been closer to 7%, as mentioned earlier in the permutation analysis. This means that the moRNA positions on the tRNA molecule do harbor information for significant identification of the ligated amino acid identity. However, our analysis shows a large difference in the cross-validated prediction power when using different partitions of the data. Partitioning by tRNA genes (t-CV) or species (s-CV) gave ∼93% prediction accuracy, whereas a partition by phylum (f-CV) lowered it to 52% or 74%. These results mean that the moRNA code found in species within one phylum does not fully reflect the code that exists in another.

If the moRNA code indeed reflects a signature used by aaRS for amino acid ligation, the results could suggest that different positions, and\or the identity of the nucleotides in these positions, may play a different role in the aaRS-tRNA recognition for species from distinct phyla. This result may also imply that the moRNA code evolved through speciation.

### Different species have a unique ensemble of codes in the informative tRNA positions

In the previous section, we showed that distinct phyla have different moRNA codes, i.e., a set of positions on the tRNA that can be used to offer high identification accuracy of the identity of their cognate amino acids (based on an out-of-sample t-cross-validation). In this section, we further explore the diversity of the moRNA code across different Archaea species. We generate the derived unique combinations of nucleotides in the eight positions that define our moRNA code in all the examined species. A total of 422 unique codes were detected. These combinations vary greatly across amino acids—from five unique moRNA codes for tRNAs translating for asparagine to 62 unique moRNA codes for tRNAs translating for arginine. Interestingly, in spite of their diversity, all moRNA codes are shared by at least two archaeal species: The average number of species sharing each moRNA is 6.7, and the most abundant moRNA code belongs to the tRNA gene sequence for methionine and is shared by 72 species.

For each species, define a species moRNA ensemble as the set of all pairs, present in that species, of moRNA code embedded in the tRNA genes and its corresponding amino acid. In our sample, these moRNA ensembles are mostly unique for each species, meaning that any two species are different for at least one amino acid in the way it is coded in the eight informative positions.

In the rare cases that two or more species share the exact same moRNA-ensemble in our data, they turned out to be monophyletic species. These are *Halobacterium* sp. and *salinarum* R1; *Methanococcus maripaludis* C6 and C7; *Methanobacterium thermoautotrophicum*, and *Methanothermobacter marburgensis* Marburg uid51637; *Sulfolobus solfataricus* and *islandicus* L S 2 15, M 16 27, M 16 4, and Y N 15 51; *Methanococcus maripaludis* C5 and S2.

Due to the uniqueness of the ensemble of the moRNA determinants among species, it may be appropriately called the *species’ moRNA cipher,* in contrast to the *universal genetic code*. This evidence strengthens the possibility that the moRNA cipher evolved through speciation.

### The divergence of the nucleotides in the informative tRNA positions coincides with the evolutionary speciation process

Since each species in our data has its own unique ensemble of moRNA codes (i.e., a combination of nucleotides in the tRNAs’ selected informative positions), it should be illuminating to study the relation between the moRNA ensembles of two species with respect to their evolutionary distance. We first define a semidistance between each pair of species by measuring the dissimilarity between their moRNA ensembles (see Supplemental Information S5). Based on the semidistances between each two species in our data set, we built a moRNA-tree (using hierarchical clustering with the “complete” linkage method). The species that are distant from each other on this tree have ensembles of moRNA code that are more “dissimilar.” We further compared this moRNA-tree with a phylogenetic tree that was created based on the *16S RNA* sequences and, therefore, entirely unrelated to the tRNA molecule and its operational RNA code (see Supplemental Information S7, S8).

**FIGURE 2.**
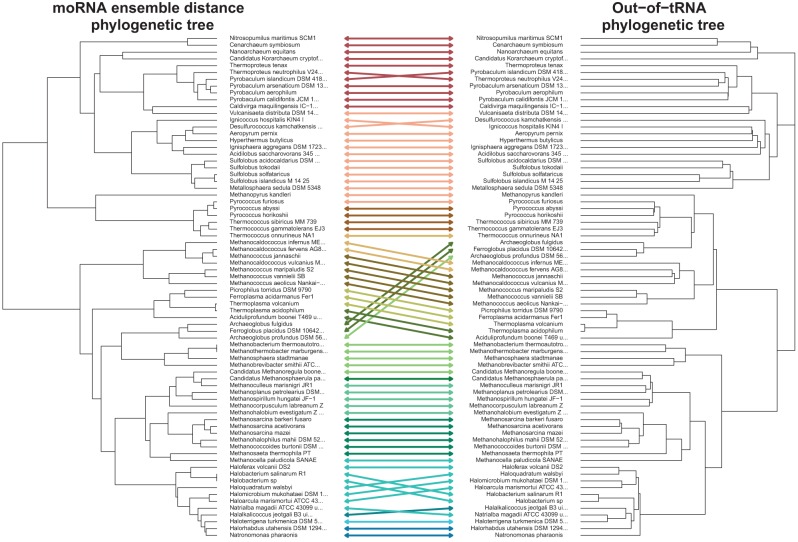
Comparison between a moRNA-tree that is based on species’ ensemble distances (*left*) and the out-of-tRNA phylogenetic tree (*right*). The out-of-tRNA phylogenetic tree is based on 16S RNA genes of 74 archaeal species (see Supplemental Information S6). The colors of the lines reflect the clusters obtained from cutting the moRNA phylogenic tree (*left*) to get 15 clusters (this number was chosen after evaluating the Bk-plot as providing a good descriptive coloring). Baker's Gamma correlation between the two trees is 0.727 (*P*-value < 0.0001). The crossing arrows indicate cases in which species were bundled in the moRNA-tree differently from the 16S-based tree. Note, however, that these crossing arrows do not allow for an interpretation of the exact evolutionary process that occurred (such as horizontal gene transfer). The significant similarity of the trees, in this analysis, might imply that the moRNA code is not conserved, but rather reflects some of the evolutionary speciation process.

The moRNA-tree, based on the pairwise distances between moRNA ensembles, is presented in Figure 2 (left). We present the phylogenic tree that was constructed for a subset of 74 (out of 86) species for which we had data to construct the 16S-based polygenic tree, presented in Figure 2 (right).

We compare these two trees using Baker's Gamma (Baker 1974) for which a value of one would mean that the two trees are topologically identical, and a value of zero would mean that the two trees are wildly different. The value in our case is 0.727 (*P*-value < 0.0001), indicating a very good statistical agreement between the trees.

In addition, a moRNA-tree based on restricting the moRNA code to its three most important nucleotide positions was built and compared with the phylogenetic tree giving a Baker's Gamma of 0.657 (*P* < 0.0001). As a sensitivity analysis of the statistical algorithm, we also compare a moRNA-tree that is based on only the three nucleotides of the anticodon to the *16S RNA* phylogenetic tree. Here we expected to get a “nonsignificant” similarity between the trees since the anticodon triplet is a functional section on the tRNA that is universally preserved. Reassuringly, we get a near-zero and nonsignificant Baker's Gamma of −0.044.

Our results suggest that the divergence between the moRNA ensemble of two species may reflect their evolutionary distance. Hence, the more two species are distant on the phylogenetic tree, the more difficult it would be to predict the identity of the cognate amino acid of the tRNAs of the one species using a model based on the moRNA code residing in the tRNA of other species.

### tRNAs for histidine and tyrosine can be distinguished from others by a single conserved position

In the previous sections, we found a subset of eight positions on the tRNA, outside the anticodon, that can adequately predict the identity of the cognate amino acid of a given tRNA gene for each of the 20 amino acids. Most aaRS (excluding Gln and Glu) are adapted to link a particular amino acid to the corresponding tRNA (Ibba and Söll 2000).

The subset of eight positions offers general insight into the information contained across the entirety of the tRNA sequences, but it does not reveal the actual number of positions, or the identity of the nucleotide residing in these positions, that are used by aaRS of tRNAs for a specific amino acid.

Therefore, we next look for tRNA positions that contain information that could potentially be used by a given aaRS to distinguish between the tRNA genes for its corresponding amino acid to the tRNA genes of all other amino acids. It is important to note that whenever a nucleotide position is found to be informative for tRNAs of a specific amino acid, and a biological interpretation is sought after, one should also consult other possible surrogate informative positions (these are captured using the corrplot in Supplemental Information S3; Supplemental Fig. S6). For example, if an analysis finds position N72 to be important, position N1 should be suspected of having equal potential for being biologically important, since these two positions often form a Watson–Crick bond and therefore contain similar information.

It could be that for tRNAs of some amino acids, even a single position contains sufficient information for (potentially) serving the purpose of aaRS-tRNA recognition. We explore this possibility by checking whether a single position (among the 43 positions) could serve as an informative position for tRNAs for one amino acid versus the rest. This is done for tRNAs of each amino acid by counting, for each position, how many times each of the four nucleotide types appears in either tRNAs for this specific amino acid or in tRNAs that do not translate to that amino acid. For instance, out of the 3936 tRNA sequences in our sample, all the 86 His tRNAs have “C” in position N73, but in the other 3850 non-His tRNAs, “C” appears in that position in only three cases. We thus conclude that, in Archaea, the informative position N73 may potentially serve as the actual discriminating position used by aaRS to distinguish tRNAs for His from other (non-His) tRNAs. (This finding is further supported by experimental evidence, as described in a later section.)

For tRNAs translating to a given amino acid, we search for an informative position that can singly discern between tRNAs for that amino acid and tRNAs for other amino acids. In other words, such an ideal position would contain one conserved nucleotide in all tRNA genes for the amino acid of interest, whereas in the tRNA genes for all the other amino acids, this position will always be occupied by one of the other three nucleotide types. This search was done by calculating the Cramer's V statistic (Cramér 1946), measuring the association between the nucleotide content for each of the 43 positions and the identity of the amino acid that is associated with that tRNA. This value measures the normalized deviation in our data from the assumption of no association. It ranges from zero, for no association, to one.

**FIGURE 3.**
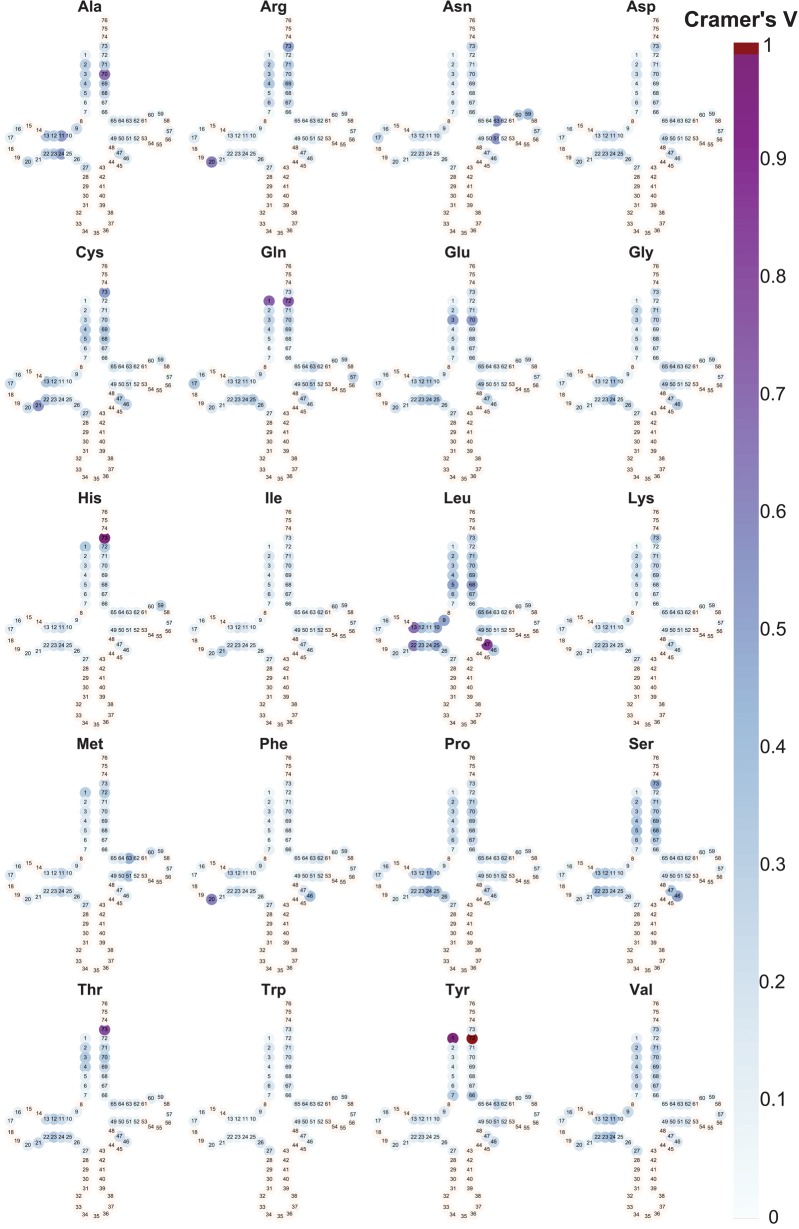
The marginal importance of tRNA positions for identifying the corresponding amino acid of tRNAs, measured by Cramer's V. Shown is the level of marginal information embedded in 43 tRNA positions for each of the 20 amino acids. A gradient of light blue to dark purple colors indicates the predicting power of a position for distinguishing the identity of a specific amino acid (from other amino acids). For example, positions N1 and N72 may be used to discern tRNAs for Tyr from tRNAs of other amino acids. tRNAs for Gln also use these positions, but they do not offer a perfect separation from tRNAs for other amino acids (therefore are not colored in dark red). In contrast, other tRNAs, such as the tRNAs for Val, have only light blue positions, indicating that no single position can be used to discern between tRNAs for Val and tRNAs for other amino acids.

A near-one value (dark purple circles in Fig. 3) indicates that a single position can, by itself, give a good prediction for the tRNA's amino acid identity—in a way that is highly preserved across all tRNA in the archaeal species used in this study. Moreover, a near-1 value of Cramer's V for a given position would imply for a high sensitivity and positive predictive value (PPV) of using that position for predicting a tRNA's cognate amino acid. To clarify terminology, the *sensitivity* of the model, for example for histidine, will be the proportion of tRNAs translating for His (tRNA^His^) that were correctly predicted as such; The *positive predictive value* (PPV) of the model, in this case, is the proportion of correctly identified tRNA^His^ out of those predicted by the model to be tRNA^His^.

The level of recognition achieved by each single position, as measured by Cramer's V value, is shown in Figure 3 for tRNAs for each of the 20 amino acids separately.

In our data set, the tRNAs for amino acids His and Tyr could be almost perfectly identified by a single position, with Cramer's V value of 0.98 and 0.99, respectively. Both have a sensitivity of one in the following way: a tRNA will (be predicted to) translate to His if it has Cytidine in position 73 (with PPV of 0.97), and Tyr if it has Guanosine in position 72 (with PPV of 0.99).

As to the tRNA genes for Leu, Thr, Gln, Ala, Arg, and Phe amino acids—we find that a specific nucleotide in one position will allow for a prediction of the tRNAs’ ligated amino acid with a high sensitivity (of almost one), but with imperfect PPV (between 0.8 and 0.4). Hence, no single position on the tRNA sequence contains enough information to allow for a full separation between the tRNAs of the desired amino acid and the others (based on the 43 positions examined, excluding the anticodon). For the tRNA genes of the other 12 amino acids, no single position has a high enough Cramer's V value (>0.6) to even suggest that a single position could be sufficient for detecting the identity of the cognate amino acid of these tRNAs. See Table 1 for details.

**TABLE 1.**
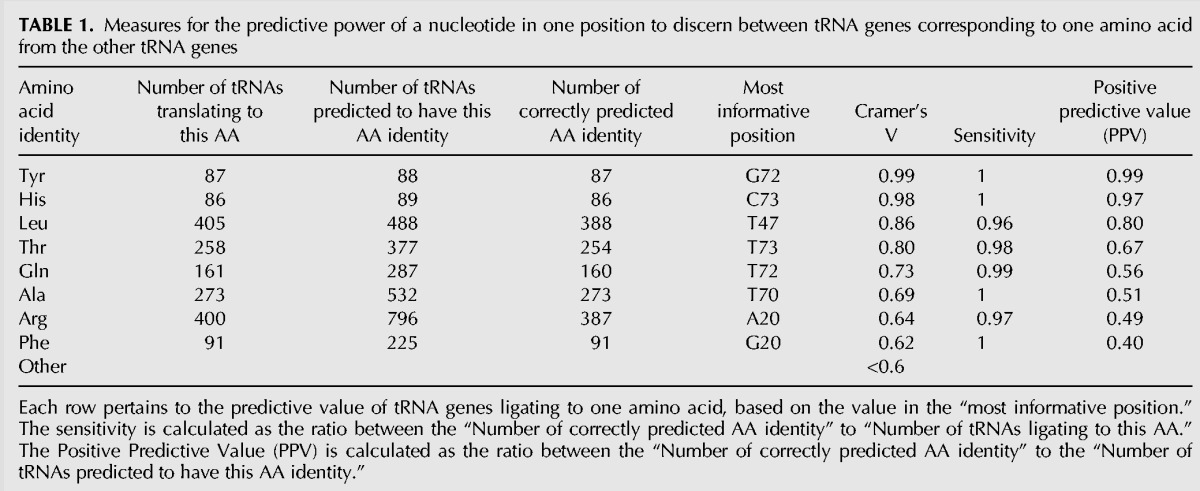
Measures for the predictive power of a nucleotide in one position to discern between tRNA genes corresponding to one amino acid from the other tRNA genes

### tRNAs for most amino acids can be distinguished from others by a small number of informative positions

In the previous section, we showed that for tRNA genes for all amino acids, except for Tyr and His, a model that could predict the identity of the ligated amino acid with a high level of accuracy (i.e., sensitivity and PPV of one) will require information from more than one position. In order to combine information from several positions for the prediction of a tRNA's ligated amino acid identity, we again use CART (Breiman et al. 1984).

The results are given in Table 2, where each row refers to the set of tRNA genes for a given amino acid, and each column (from left to right) depicts an increasing number of positions used by CART for predicting the tRNAs’ cognate amino acid identity. For example, the cell in the first row (“Ala”) and the column “X2” presents the prediction accuracy of identifying tRNAs for alanine when using a model that relies on two positions. Notice that the column “X0” refers to the proportion of tRNA sequences not translating for Ala (i.e., the prediction accuracy of using no position and just predicting that all tRNAs are not ligated by Alanyl-tRNA Synthetase). The “Nucleotide identity (ordered)” column shows the identity and order in which positions are added sequentially to the model.

**TABLE 2.**
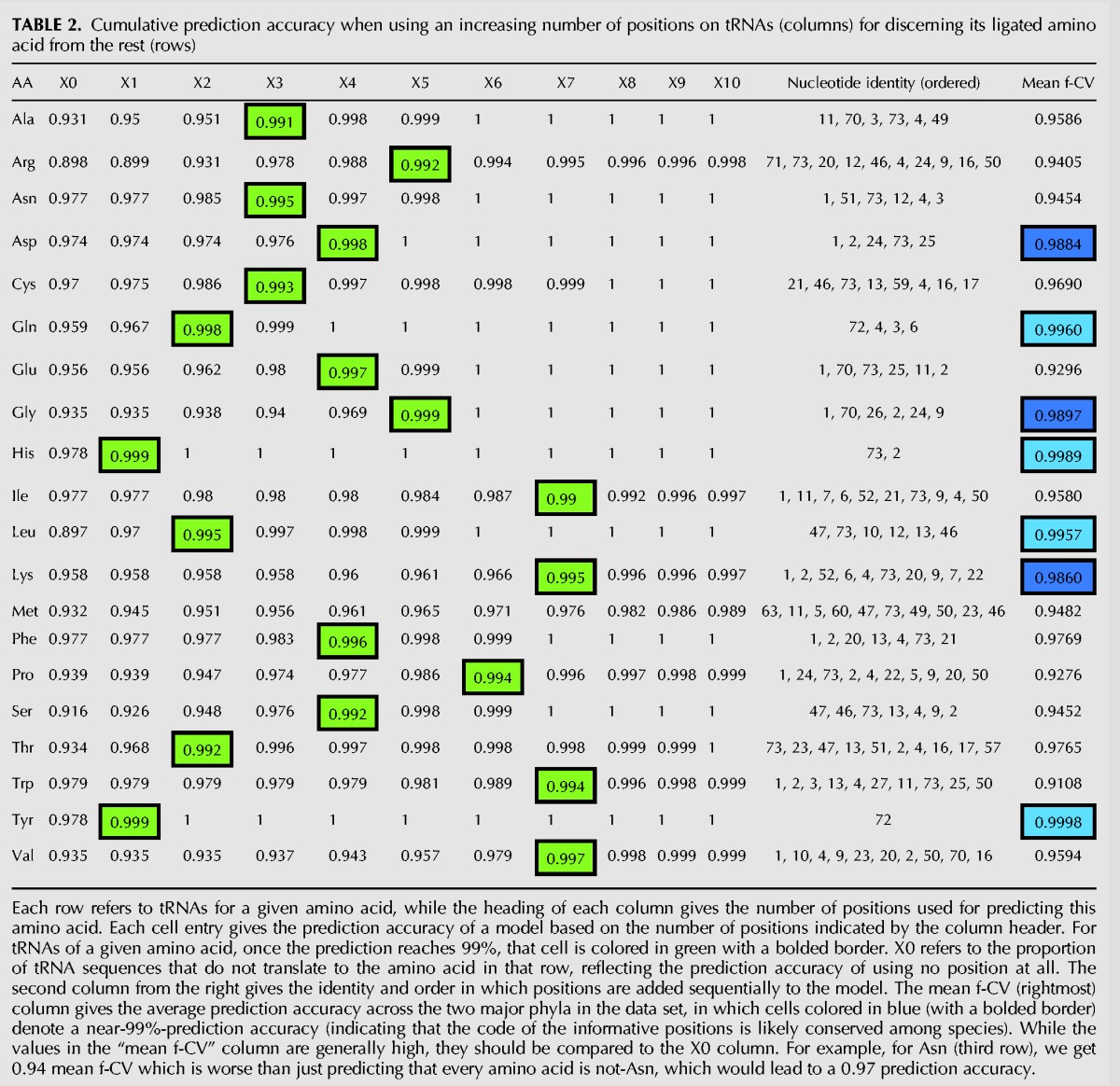
Cumulative prediction accuracy when using an increasing number of positions on tRNAs (columns) for discerning its ligated amino acid from the rest (rows)

In order to reach 99% prediction accuracy, a model that discerns the identity of tRNAs for His from tRNAs for other amino acids can rely on a single position, as expected from our previous analysis. A single position is also sufficient for discerning between tRNAs for Tyr from that of tRNAs for other amino acids. A model for discerning between tRNAs for each of the other 18 amino acids (compared to the rest) will require more than one position. tRNAs for each of the Gln, Leu, and Thr amino acids will require two positions to be distinguished from tRNAs for all other 19 amino acids. tRNAs for Ala, Asn, and Cys require three positions; tRNAs for Met require 10 positions. The tRNAs for the remaining 11 amino acids require 4–7 positions to discern the identity of the corresponding amino acid of each from that of the other tRNAs.

An intriguing question is how common is the tRNA code (for discerning tRNAs for one amino acid from tRNAs of all other amino acids), in the discovered positions, across different archaeal species. To answer this question, (i) the model of minimal size to achieve within sample prediction accuracy of 99% with tRNA genes of one phylum (Euryarchaeota or Crenarchaeota) was found, (ii) it is used to predict the identity of the ligated amino acid of tRNA genes from the other phylum, and (iii) the resulting average out-of-phylum accuracy (per amino acid f-CV) is reported.

The results are given in the rightmost column of Table 2. The cells colored in blue denote a near-99% prediction accuracy (of per amino acid f-CV), indicating that the set of positions and code therein, found by our model, is reasonably conserved across the two phyla (i.e., contains similar information across phyla that allows for a good prediction of the identity of each tRNA's ligated amino acid).

As expected, in the case of tRNAs for His and Tyr, there is one conserved position across phyla—containing information for discerning tRNAs for either His or Tyr amino acids from tRNAs for the other 19 amino acids. As to the tRNAs for Leu and Gln, two informative positions are found to be conserved. The tRNAs for Asp, Gly, and Lys might have somewhat conserved positions across phyla (near-99% prediction), and require more than three informative positions. The tRNAs for the remaining amino acids display a clear indication that the code in their informative positions is not conserved across phyla. Alternatively, it could mean that the needed information is located somewhere else—either in the anticodon or in some other position(s), not represented in the current data set.

Altogether, our results suggest diversity in the number of positions needed for the (potential) recognition of some tRNAs by their corresponding aaRS in the Archaea domain. tRNAs for four amino acids require the same one (His, Tyr) or two (Gln, Leu) informative positions and tRNA code across all archaeal species, while others require more positions, and their tRNA code does not extrapolate across phyla.

### Experimental evidence supports the statistically derived informative tRNA positions and code

In the previous section, we identified a set of informative positions on tRNAs for each amino acid that could *potentially* mediate the recognition between a tRNA and its corresponding tRNA synthetase. In this section, we compare the statistical results with experimental evidence on the identity of such positions. Reassuringly, we found a strong agreement between the statistically derived positions’ importance and the previously published empirical results.

We found that a single informative position (with a Cramer's V of 0.99) is sufficient for discerning between either tRNAs^His^ (using N73) or tRNAs^Tyr^ (using N72), and tRNAs for all other amino acids—for all archaeal species in our data. For tRNAs^His^, it was experimentally shown (see Table 3) that *Aeropyrum pernix K1* uses position N73 (Nagatoyo et al. 2005). Regarding tRNAs^Tyr^, it was found that three positions (other than the anticodon) are assumed to participate in the recognition process: N72, N1, and N73 (Fechter et al. 2001; Iwaki et al. 2002, 2012; Tsunoda et al. 2007). This is in agreement with our results where positions N72 and N1 are found to be highly informative (both contain nearly the same information due to their Watson–Crick bonds). Our model did not detect position N73 as containing additional information beyond what is found in either N1 or N72 for the recognition between tRNAs^Tyr^ and its corresponding aaRS. Furthermore, our results are consistent with the crystal structure analysis of *Methanococcus jannaschii* TyrRS (Kuratani et al. 2006), indicating that Ile15 in the *cis* peptide bond of the TyrRS interacts with position N1 of the tRNA^Tyr^.

**TABLE 3.**
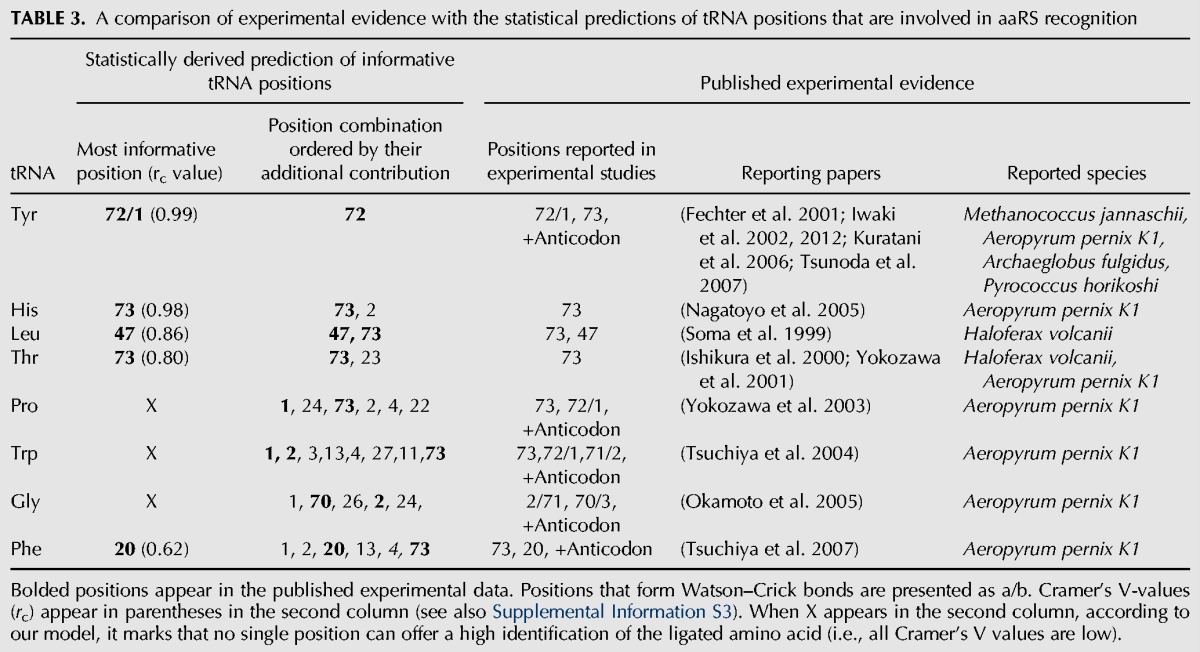
A comparison of experimental evidence with the statistical predictions of tRNA positions that are involved in aaRS recognition

Two informative positions are sufficient for statistically discerning each of the tRNAs^Leu^ and tRNAs^Thr^ from tRNAs for all the other amino acids (across all archaeal species in our data). For tRNAs^Leu^, it was experimentally shown that *Haloferax volcanii* uses positions N73 and mainly N47 (Soma et al. 1999), as was also found in our analysis. For tRNAs^Thr^ it was shown in three studies (Ishikura et al. 2000; Yokozawa et al. 2001) that position N73 participates in the aaRS recognition process. Reassuringly, this position is one of the two positions detected by our model. However, our data set suggests that position N73 alone does not contain enough information to uniquely identify tRNAs^Thr^ and that another position, for example N23, is required. We are not familiar with experimental evidence of important positions for aaRS recognition of tRNAs^Gln^ in Archaea, while our model located two conserved informative positions (N72, N4).

Our statistical analysis showed that more than three positions are needed for proper prediction of the amino acid identity of tRNAs translating to Pro, Trp, Gly, and Phe. Furthermore, our f-CV analysis on tRNAs for these amino acids indicates that species from different phyla have different sets of positions and/or tRNA code that carry the information that (potentially) allows for proper discerning by their respective aaRS. For tRNAs^Pro^, our analysis identified positions N1 and N73 as informative positions, consistent with published reports, but also found four other positions that were not reported. For tRNAs^Trp^, we identified N1 and N2 as informative positions in agreement with the published experimental results. However, N73, also found experimentally, was not required by our model (given five other positions). For tRNAs^Gly^, both the experimental studies and our model found N70 and N2 as important. As to tRNAs^Phe^, we recognized position N20, in agreement with the experimental studies, but position N73 (which was also experimentally found as important) was not needed by our model (given the other positions already used by the model). Hence, our analysis not only detected most of the positions outside the anticodon that were experimentally shown to be important in the literature but also detected other potential positions that were not reported in the literature.

Overall, our predictions are highly supported by experimental evidence. Interestingly, for tRNAs of amino acids that our model detected as having one or two informative positions and tRNA code used by all the 86 archaeal species, our predictions were fully aligned with what was found in the literature. Moreover, as expected, for tRNAs of amino acids that we detected as associated with different sets of positions and code across phyla (i.e., not conserved across species), the positions we found in our analysis (for all 86 species) overlapped only partially with the results in the literature that were based on only one archaeal specie (*Aeropyrum pernix* K1).

## DISCUSSION

In this study, we used statistical analysis to identify potential information along the tRNA sequences, characterized by specific nucleotide positions and identity, which may serve for the recognition between the tRNA and its corresponding aminoacyl tRNA synthetase. Our analysis bundled together tRNA genes translating all the codons corresponding to a specific amino acid; it is not expected to pinpoint sequence elements that are associated with a specific anticodon. Similarly, as our analysis aims to identify nucleotide positions that reflect discerning between tRNAs for a given amino acid and tRNAs for all other 19 amino acids, it will ignore nucleotide positions and identities that are common among all or most tRNA genes (i.e., global tRNA sequence elements). The life cycle of a tRNA molecule is complicated, as it requires transcription and further processing, including base modification, before charging with an amino acid. Tens of different tRNA modifications are known; in fact, a study by Sprinzl (1998) indicates that over 10% of the residues in several species are subjected to modifications. Our analysis focuses on the identity of the nucleotides in the various positions, but it does not aim to reach further information about potentially associated post-transcriptional modification.

The set of positions, and nucleotides within, found by our analysis does not seem to serve as a common tRNA code to all Archaea species. This is reflected by the observation that distinct phyla have their own signature at the informative positions. Even across species of the same phylum, there is variability in the moRNA ensembles of informative tRNA codes. A more refined look at tRNA genes for each of the 20 amino acids revealed that tRNAs for only four amino acids (namely, His, Tyr, Leu, and Gln) use, in the same informative positions, a common tRNA code—across all the archaeal species in our data set. This may imply a common tRNA code, embedded in the tRNAs for these amino acids, across all archaeal species. However, conserved nucleotide content in specific positions, in the sequences of tRNAs for the same amino acid among different species, will not necessarily be enough to define a tRNA code and may even not be a part of it at all. For instance, the pair G3:U70 is almost universally conserved in alanine tRNA genes across all three domains of life (Ribas de Pouplana and Schimmel 2001), yet our analysis demonstrates that these two positions alone cannot fully distinguish between tRNAs for alanine amino acid and tRNAs for other amino acids (i.e., perfect sensitivity but nonperfect PPV). This suggests the potential role of additional positions, perhaps in the anticodon itself, in the prevention of tRNA misloading.

Moreover, a conserved informative position in tRNAs for some amino acids of species from the Archaea domain does not necessarily indicate that the same tRNA code would be used in species from other domains. For example, published work on the role of the paired positions N2:N71 in tRNAs for the aminoacylation of tyrosine shows that while bacteria's aaRS requires the G:C nucleotides pair in these positions for proper aminoacylation, such a structure does not enable the aminoacylation of tyrosine in humans or Archaea. Yet the simple switch to C:G in N2:N71, in the tRNA, does enable this species-specific aminoacylation (Quinn et al. 1995; Wakasugi et al. 1998; Tsunoda et al. 2007). In our study, we found that Archaea species contain the relevant information in positions N1:N72, and not N2:N71 (as it was also found experimentally, see Table 3.). Other experiments (Wakasugi et al. 1998) showed that the switch from G:C to C:G was achieved by a simultaneous change in the amino acid composition of the tRNA synthetase's domain that adapted to N2:N71 recognition (Ribas de Pouplana and Schimmel 2001). Such transitions in coding mechanism, while using the exact same positions, would be harder to detect when analyzing species from different domains using the same statistical model, but may be mitigated by analyzing each kingdom separately. At the same time, some patterns may be preserved across domains and are consistent with our conclusions regarding the Archaea species. For example, it was shown in vivo and in vitro that C73 is a discriminator nucleotide for tRNA^His^ in *Escherichia coli* (Francklyn and Schimmel 1990; Yan and Francklyn 1994). This was shown in experiments as well as in our analysis for tRNA^His^ in Archaea.

An attractive interpretation of our results is that the code embedded in the ensemble of tRNAs’ informative positions may be viewed as a *species-specific evolving cipher*, unknown in its entirety to any single other species. As opposed to the universal DNA code that is, like the Morse Code, known to all, a cipher encrypts a message, offering protection of its content from onlookers. As an analogy, one may think of each moRNA code on the tRNA sequence as a key, with the ensemble constituting a ring of keys. Each species has its own unique ring of keys, used for unlocking a critical part of the translation machinery (i.e., the proper ligation of a tRNA with its cognate aaRS). Each ring of keys is unique, even though the individual keys on the ring (for some amino acids) may be shared among different species. Furthermore, the more two species are evolutionarily distant, the less similar are their rings of keys. Hence, even when making a comparison within the same domain, the further apart two species are on the domain's phylogenetic tree, the less we can infer from their “ring of keys” (informative positions for tRNA-aaRS recognition) of one species about the combination of “keys” used by the other species.

When considering potential post-transcriptional modifications on different positions and nucleotides along the tRNA molecule, it is clear that this tRNA code probably displays a higher level of complexity that gives room for more potential regulation of aminoacylation. Recent studies (in prokaryotes and eukaryotes) demonstrated not only noncanonical roles of uncharged tRNA molecules in the cell, but also that of aminoacyl-tRNAs. Besides their function in protein biosynthesis, it now turns out that aa-tRNAs participate in various biochemical processes, such as cell wall formation, protein labeling for degradation, aminoacylation of phospholipids in the cell membrane, and antibiotic biosynthesis (Raina and Ibba 2014). Is there a correspondence between the variations in recognition elements along the tRNA sequence to other sequence motifs associated with other biological processes? Our current study focuses on identity elements that are associated with aaRS-tRNA recognition, yet it is tempting to hypothesize that the existence of different combinations of tRNA-aaRS recognition elements may serve for selective charging of specific sets of tRNA genes with their cognate amino acid in either a condition- or time-dependent manner.

While we can find a set of informative positions, our analysis cannot distinguish which of these nucleotide positions plays a functional role in the aminoacylation process itself, in terms of direct physical interaction. Furthermore, if an informative set of tRNAs for some specific amino acid contains many positions, it does not necessarily mean that the interaction between the tRNA and the corresponding aaRS relies on all these positions. It is possible that different species use combinations of positions that are slightly different from the ones discovered in our model or that the information used by the aaRS is located also outside the scope of the 43 positions we examined, possibly including the anticodon itself. Whether positions outside the anticodon are used independently of the anticodon, or merely support it for correct aaRS-tRNA recognition, remains to be verified experimentally. For example, it is known that the anticodon is indeed important for the aaRS recognition by 17 of the 20 *Escherichia coli* iso-accepting groups; whereas for tRNAs translating to the three remaining amino acids, the ligation process does not rely on the anticodon (Saks et al. 1994). It could be, as well, that a different combination of positions (one not discovered by the greedy search used in this work) would give an alternative code. For example, when our analysis finds an informative position that is Watson–Crick bound to another position on a stem, it cannot pinpoint which of the two positions is crucial for the tRNA-aaRS recognition in the cell.

The interpretation of the informative positions (outside the anticodon) and the code therein as an evolving cipher suggests that they may play some role in the speciation process itself, rather than merely reflecting it. Such an evolutionary speciation process is expected to be reflected in the co-evolution of the corresponding aminoacyl tRNA synthetases’ genes in the same species. Indeed, some experimental work shows the co-evolution of tRNAs with their corresponding aminoacyl tRNA synthetases’ genes (Salazar et al. 2003), and there is room for more such research. In the context of previous works showing the limitation in amino acid ligation when using tRNAs from species of different domains (Ripmaster et al. 1995; Kobayashi et al. 2003; Namgoong et al. 2007; Shaul et al. 2010), our results may suggest the existence of informative tRNA positions and code which are species-specific, across all the three domains of life. Our method of analysis for searching potential determinants of selective tRNA aminoacylation could be implemented for other species/kingdoms and may further direct relevant experimental research. In this work, we focused on exploring the different concepts and tools in the Archaea kingdom, for which a much further understanding of the tRNA code is needed. Our methods and strategies can be further implemented for the future study of the tRNA code within and across the other domains of life.

Lastly, since aminoacyl-tRNA synthetase inhibitors are known as potential targets for antibiotics (Vondenhoff and Van Aerschot 2011; Agarwal and Nair 2012), once a unique cipher is experimentally verified, the tRNA cipher information may be used for the development of antibiotics that are targeted against specific species while keeping their host unharmed.

## MATERIALS AND METHODS

### Data acquisition

The tRNA data set was obtained from the output of the tRNAscan-SE program, making 3936 tRNA gene predictions on complete or nearly complete genomes of 86 archaeal species, as was available for extraction from the “genomic tRNA database” (gtrnadb) website (Chan and Lowe 2009).

The web pages of the site were downloaded, and metainformation for each tRNA was obtained and prepared by web-scraping of the site using R (Hadley 2007; Wickham 2011, 2015; Temple Lang 2013; R Core Team 2014; Lang and the CRAN Team 2015). The extracted metainformation includes, for each tRNA, its primary structure, predictions for introns in the sequences, secondary structure folding, and the exact location of the anticodon. Further details are available in Supplemental Information S1.

### Sequence alignment

The classical model for a tRNA primary structure of the tRNA molecule is often 76 nt in length (Sprinzl et al. 1972; Kim et al. 1974; Giegé et al. 2012), including the CCA end tail of the molecule. However, in their raw form, the extracted tRNA sequences we have in our data set deviate from this model. The process of cleansing and aligning the sequences involved the following four steps (more details are given in the Supplemental Information): (i) finding and removing the “CCA” tail ending; (ii) removing introns from the sequences, as detected by the tRNAscan-SE Genomic tRNA Database (Chan and Lowe 2009); (iii) positions gaps were added. Based on the tRNAscan-SE algorithm, the aligned sequences were inspected and gaps were added based on the anticodon location, and the positions of the stems and of known conserved positions; and (iv) extracting candidate nucleotide positions for the generalized operational RNA code. Each of these steps is detailed in Supplemental Information S2.

### The classification tree algorithm (CART)

A decision tree is an approach for discovering relationships among explanatory variables and a dependent (outcome) variable for either prediction and/or understanding. CART is an algorithm for the construction of a decision tree by recursively partitioning the space of the data by asking an ordered sequence of binary (yes or no) questions on it. The question asked at each subsequent step depends on the answers to the previous questions of the sequence. Once the sequence of questions terminates, it offers a prediction of the outcome. CART is a “greedy” algorithm that does not attempt to optimize the performance of the whole tree; and by restricting itself to a one-step look-ahead strategy, the CART method avoids the combinatorial explosion of splitting options that occurs when trying to search the entire space of potential trees, while accepting the risk of finding only a suboptimal tree.

The CART algorithm (Breiman et al. 1984), as implemented in the *rpart* package (Therneau and Atkinson 1997), was used to generate the rules identifying the ligated amino acid from the generalized operational RNA code determinants.

The tree was fully grown with no penalty for its size, using the information impurity measure to choose between potential splits. The identification accuracy of the model is defined as (1 − “misclassification error”), with the “misclassification error” defined as the ratio of the number of misclassified amino acids to the number of generalized “operational RNA code” determinants.

The search for a smaller subset of nucleotide positions was conducted using a (greedy) forward selection method: starting from step one, the best single nucleotide position was selected for building an amino acid identification model; at each forward step the nucleotide position adding most to the cumulative identification accuracy was added, and a new CART model was fitted.

A visualization of nucleotide frequencies of the moRNA cipher is presented in Supplemental Figure S9.

### Model validation

The predictive accuracy of a classification tree, assessed by relying on the same sample that was used to build it, gives the within-sample (resubstitution) prediction accuracy. To assess how the results from the classification trees that we constructed generalize to new data, it is important to capture the biological constructs contributing to the variance in our data. Therefore, four variations of the cross-validation method were used:

- In the *t-RNA cross-validation* (t-CV), we take 100 random samples of our species (each time using 66.7% of our tRNA sequences). For each training sample, we build a classification model from, and test its identification success (of the amino acid identity of each tRNA) on, the tRNAs of the remaining tRNAs.
- In the *species cross-validation* (s-CV), our set of species was randomly partitioned 100 times into 66.7% of the species in the training set and the rest for the validation set (keeping the entire ensemble of tRNAs of a species either in one part or in the other). The identification successes of the tRNAs’ amino-acid identity on the validation sets are averaged.
- In the *family cross-validation* (f-CV), our set of domains of Archaea was partitioned so that the model was built on one family and its accuracy tested on the other; accordingly, the identification success reflects the generalizing power to new families. This was done twice.

At each repetition of t-CV, s-CV, and f-CV, the eight nucleotides’ positions that comprise the moRNA code were chosen using the training set only.

Assessing the magnitude of the identification success of our model is done through a permutation test. Keeping the determinants constant, the identity of the ligated amino acids is randomly permutated, and a new model is fitted. The resubstitution identification success and the 10-fold CV were calculated. These two were averaged over 50 permutations.

### Distances between species based on tRNA ensembles

Measuring the dissimilarity between a pair of moRNA ensembles (i.e. species) is done in a hierarchical fashion by: (i) counting the number of nucleotide positions that differs between pairs of moRNAs that correspond to the same amino acid, one from each ensemble; (ii) then summing the counts over all such possible pairs per amino acid; and and lastly (iii) , averaging the distances over all amino acids of the two species. Clearly, if two moRNA ensembles (species) are identical, the distance is zero, and vice versa. For other properties of this measure, which is theoretically a semidistance and, practically, a distance in our data, see Supplemental Information S5.

### Constructing phylogenic trees

For a given distance between species, based on a subset of nucleotide positions from moRNA, a phylogeny tree of the species was built using a simple agglomerative procedure that relies on the complete linkage method (using hclust in R).

The “traditional” phylogenetic tree is based on 16S RNA genes from 74 archaeal species. Alignment was done with MEGA, with unalignable parts deleted. The tree is a neighbor-joining one with 200 bootstraps. We are left with 74 out of 86 archaeal species for the phylogenetic tree analysis because the species lists are incompatible between the public databases that we used.

The two rooted phylogenetic trees were rotated and drawn opposite to each other, using auxiliary lines to connect same taxa, in a tanglegram plot (Fig. 2). The code for tree manipulation and comparison using the tanglegram plot was created using the *dendextend* R package (Galili 2015).

### Measuring the similarity of two phylogenetic trees

For the comparison of two phylogenetic trees, we use the gamma index introduced by Baker (1974), defined as the rank correlation between the stages at which pairs of objects combine in each of the two trees. Since the observations of such a measure are correlated, we used a permutation test for the calculation of the statistical significance of the index, randomly reshuffling the leaf labels on the moRNA-generated tree and comparing it to the outside tRNA tree using Baker's index. This process was repeated 1000 times, and the following quantiles were computed: q_.025_ = −0.08 and q_.975_ = 0.109; q_.005_ = −0.095 and q_.995_ = 0.146. An alternative method called the Bk plot is presented in Supplemental Information S8. These calculations were performed using the *dendextend* R package (Galili 2015).

We also considered using the Robinson–Foulds distance, but because it is well defined only for bifurcating trees, and since our trees include segments that are not binary splits, we chose to go with a more robust metric that is not sensitive to the number of splits, namely Baker's Gamma correlation.

## SUPPLEMENTAL MATERIAL

Supplemental material is available for this article.

## Supplementary Material

Supplemental Material
