# Supplementary material for: Identifying the ligated amino acid of archaeal tRNAs based on positions outside the anticodon
Source: RNA. 2016 Oct;22(10):1477–91. doi: 10.1261/rna.053777.115 (PMC5029447; doi:10.1261/rna.053777.115)
Supplement: Supplemental Material [file supp_22_10_1477__index.html]

Identifying the ligated amino acid of archaeal tRNAs based on positions outside the anticodon — Supplemental Material 

# Identifying the ligated amino acid of archaeal tRNAs based on positions outside the anticodon

## Supplemental Material

- Supplemental\_Information.doc
