# Supplementary material for: Identifying the ligated amino acid of archaeal tRNAs based on positions outside the anticodon
Source: RNA. 2016 Oct;22(10):1477–91. doi: 10.1261/rna.053777.115 (PMC5029447; doi:10.1261/rna.053777.115)
Supplement: Supplemental Material [file supp_053777.115_Supplemental_Information.doc]

Supplementary Information

Sections:

1. Data acquisition

2. Sequence alignment

3. The association between nucleotides at pairs of tRNA positions

4. The use of the CART algorithm

5. Distances between species based on tRNA ensembles

6. Constructing a phylogenic trees

7. Measuring the similarity of two phylogenetic trees (Baker's gamma)

8. (K, Bk) plots –definitions and illustrations

9. Visualizing the moRNA cipher

10. References (extended)

Figures: S1-S11

## 1. Data acquisition


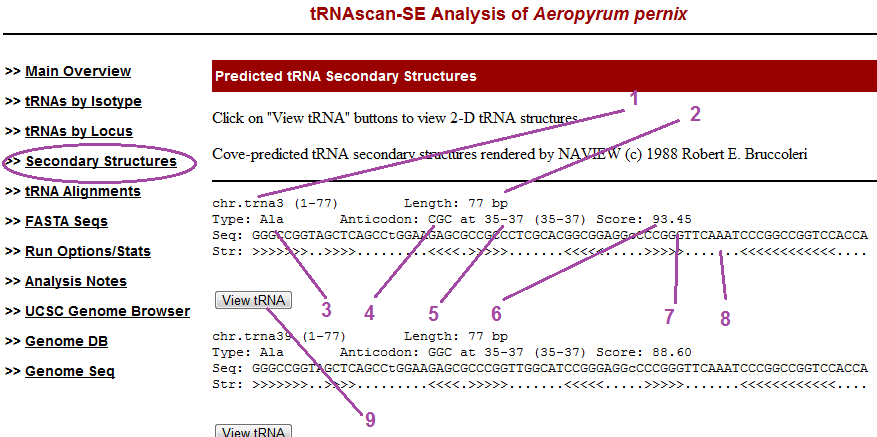


*Fig. S1: An example of Aeropytum pernix “>> Secondary Structures” tRNA page, with indication of each of the elements that will be extracted. (The screenshot was taken from* [*http://gtrnadb.ucsc.edu/Aero_pern/Aero_pern-structs.html*](http://gtrnadb.ucsc.edu/Aero_pern/Aero_pern-structs.html)*)*

Figure notation:

1. **Chr.trna*** - tRNA id number
2. **Length** - The length of the tRNA molecule (including introns)
3. **Type** - The amino acid that corresponds for this tRNA (based on the tRNA’s anticodon)
4. **Anticodon** - The anticodon sequence
5. **At (*-*)** - The position of the anticodon
6. **Score** – The "Cove score" of that tRNA. The higher the score, the better the tRNA fits the traditional secondary structure model
7. **Seq** - The tRNA sequence. Note that nucleotides matching the "consensus" tRNA model used in Cove analysis appear in upper case letters, while introns and other nucleotides in non-conserved positions are printed in lower case
8. **Str** - Contains the predicted secondary structure folding of the tRNA, with nested ">" and "<" symbols representing base pairings. Explanation for its structure is provided at <http://gtrnadb.ucsc.edu/legend.html>
9. **“View tRNA”** – Visualizes the secondary structure of that tRNA


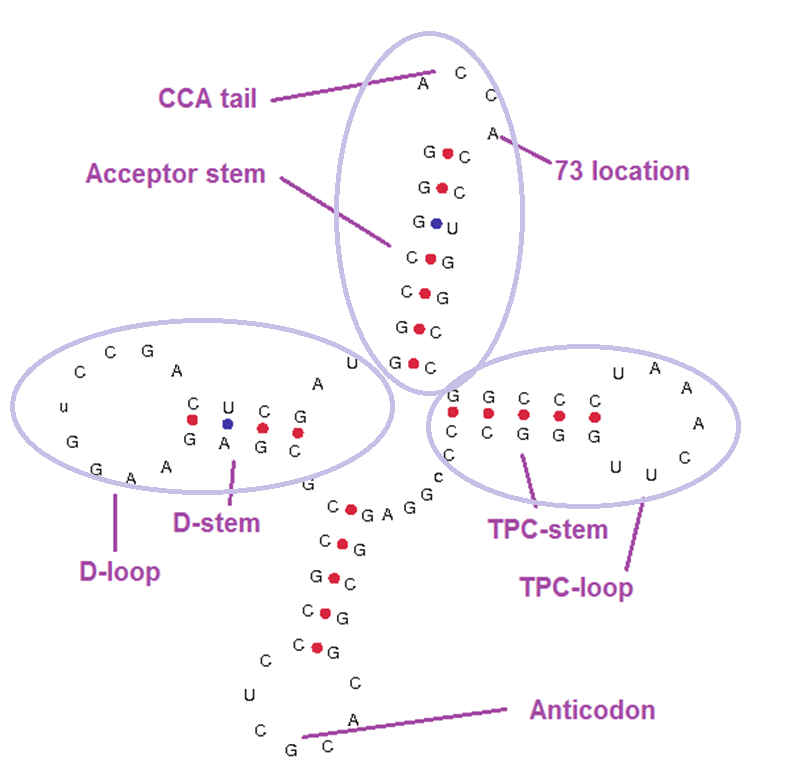


*Fig. S2: An example of tRNA secondary structure diagram for Aeropytum pernix, with annotations of the various regions. (Source: a screenshot (+notations) from* [*http://lowelab.ucsc.edu/cgi-bin/draw_trna.cgi?%20chr.trna3*](http://lowelab.ucsc.edu/cgi-bin/draw_trna.cgi? chr.trna3)*)*

## 2. Sequence alignment

**Step 1: finding and removing the “CCA” tail ending.**

The CCA tail is a cytosine-cytosine-adenine sequence at the 3' end of the tRNA molecule. This sequence is mediate the acylation of the tRNA with an amino acid, hence is critical in the translation process. In prokaryotes, the CCA sequence is sometimes not present in the gene of the tRNA and is added only during the creation (processing) of the tRNA.

Since this “CCA” N74-N76 extension of the molecule is available only in some of the tRNAs in our dataset, in order to have comparable sequences of tRNA molecules normalized to the typical 73-sequence length, the CCA sequence was removed whenever present.

We were able to detect the CCA tails based on the fact that most tRNA's acceptor stem and TΨC arm retain a high level of Watson-Crick bonds. The notation of the secondary structure for the positions from 60 to 73 should often be ".<<<<<<<<<<<<.". This is true for 71.9% of the tRNAs in our dataset. Of the remaining sequences, 25.6% were of type ".<<<<<<<<<<<<...." , including an extra CCA ending to the molecule, and all such cases had their extra CCA tails trimmed as well. With the remaining 2.5% of tRNAs, we found 36 variations of the secondary structure of the last 17 nucleotides. The decision on when to remove the CCA ending was made individually on each of the variations, depending on whether or not it had 4 dots at the end, and the number of “<” throughout the string.

**Step 2: Removing Introns (and other “non-conserved” positions) from the sequences.**

The tRNAscan-SE Genomic tRNA Database gives “On the sequence line, nucleotides matching the "consensus" tRNA model used in Cove analysis appear in upper case, while introns and other nucleotides in non-conserved positions are printed in lower-case letters.” (source: <http://gtrnadb.ucsc.edu/legend.html>).

Since our interest is in modeling the structure of the mature tRNA molecule, we used these predictions in order to remove all possible introns (or other possible “non-conserved positions”) from both the primary (“Seq”) and the secondary (“Str“) sequences (i.e.: the lower-case letters).

After the second stage, almost 60% of our tRNAs had 72 nucleotide positions, about 20% had 71 positions, while about 20% of the tRNAs had 80-82 nucleotide positions.

**Step 3: Locating gaps based on the location of the anticodon, the stems, and conserved positions**

The sequences we work with are all aligned using the tRNAscan-SE Detection Algorithm (see: <http://gtrnadb.ucsc.edu/legend.html>), which offers a secondary structure for the sequences using a tRNA covariance model.

Based on the “consensus” tRNA model, the anticodon should start at position 34. This is indeed the case for 74.5% of the sequences. However, we found that 25.5% of the sequences (1017) were (likely) missing one nucleotide position (“gap”) between positions N1 and N33 of the molecule, resulting in the anticodon starting at position N33 (instead of N34).

Before adding gaps, we first simply stacked the sequences on top of each other, and plot the nucleotide frequency at each position to detect conserved areas across the sequences (see logo plot in Figure S3).

*Fig. S3: A sequence logo plot for positions 1 through 33 (the region upstream to the anticodon) for all sequences*

We wish to identify the missing gap for the 25.5% of our sequences where the anticodon starts in position 33 instead of 34.

Before adding the missing gap, and using information from the secondary structure from the covariance model, the sequence logo plot reveals that positions N8, N14, N15 and N18 are highly conserved (with the nucleotides T, A, G and G, respectively). This conservation is consistent with the consensus sequence of the A-box (Ciliberto, Raugei, Costanzo, Dente, & Cortese, 1983; Marck et al., 2006). This tells us that most of the 25.5% of the sequences with a missing gap should be located somewhere after position N18 (since if the gap was before N18, it would “contaminate” the conserved positions, for example – N14 would have many G’s and not only A’s).

Whenever we had the anticodon start on position 33 instead of 34, we sequentially checked whether the conserved positions contain the "correct" (conserved) nucleotide. If not, a gap is added in the location of the conserved nucleotide, starting with position N8, then N14, N15, N18, and lastly position N19 (when the gap is added in a conserved position, for the purpose of plotting, it was replaced with the conserved nucleotide of that position. This temporary imputation method is fine since these positions are later removed entirely for all subsequent analyses). This was done also while considering the Watson-Crick bonds in the secondary structure (which was detected using the covariance model).

For 16 tRNA sequences we added a gap in position N8 since they did not have the conserved letter T, and since their nucleotides in positions N10-N13 (of the D stem) did not have a matching Watson-Crick bond with positions N22-N25 (if they had, we would know that there was no missing gap, but more likely, a misplaced nucleotide in that position).

For 18 tRNA sequences we added a gap in position N14 since they did not have the conserved letter A, and since their nucleotides in positions N10-N13 (of the D stem) did not have a matching Watson-Crick bond with positions N22-N25 (if they had, we would know that there was no missing gap, but more likely, a misplaced nucleotide in that position, and that the gap was somewhere else).

For 41 tRNA sequences we added a gap in position N15 since they did not have the conserved letter G (again, considering the alignment of the D stem).

For 4 tRNA sequences we added a gap in position N18 since they did not have the conserved letter G (again, considering the alignment of the D stem).

For 983 tRNA sequences we added a gap in position N19 since they had the correct conserved positions in N8, N14, N15 and N18 while they did not have a proper Watson-Crick bond (as given in the secondary structure) of the D stem. Hence, the D loop (likely) had only 7 positions instead of 8 and a nucleotide is missing in the D loop in either positions N19, N20 or N21. All of the 74.5% of sequences for which the anticodon started in position N34 had the nucleotide G in position N19, hence (for the purpose of plotting the sequence logo plot of Fig S4), we imputed this gap in N19 as the letter G (again, this was later removed for the subsequent analyses).

For similar reasons, a gap was added in position N24 to 5 tRNAs.

This process has resolved almost all of the misplaced anticodon positions, leaving only 0.3% sequences (15) for which the anticodon starts before position 34.

The updated sequence logo plot (Fig S4) displays the results of the alignment process. It is reassuring to see that this addition of a gap created another conserved position in N33 (having the nucleotide T in Fig S4, which did not show in Fig S3).

*Fig. S4: A sequence logo plot for positions 1 through 33 for all tRNA sequences, after adding nucleotides in conserved positions*

Using a similar strategy for the sequences downstream to the anticodon resulted in (almost) no need for adjustments (gap adding). For 3,885 sequences, we had a (nearly) perfect Watson-Crick pairing (based originally on the tRNA covariance model) of positions N65-N61 vs N49-N53, while the conserved positions had the conserved nucleotides (N61=C ; N56,N55,N54=CTT ; N53=G).

*Fig. S5: A sequence logo plot for positions N73 through N44 for all sequences*

**Step 4: Extracting candidate positions for the goRNA**

15 non-conserved nucleotides were chosen from the middle, 15 from the 5', and 13 from the 3' of the tRNA molecule, as candidates for the goRNA code.

The remaining (unused) positions are: N8,N14,N15,N18,N19,N33,N48,N53,N54,N55,N56,N58,N61,N43.

We wanted to have as many nucleotides as possible for the analysis, while making sure we do not use nucleotides which are too close to the anticodon region. For this reason we made sure that we are taking from the 5' end all the nucleotides from position number N1 until position N27. In the 3' end, we made sure that we are taking all the nucleotides from N73 until N46 (excluding the conserved nucleotides). We stopped at N46, because starting from N45 and onward, it is not clear how to define N45, due to the existence of the variable arm. When the variable arm is present, which seems to happen only for the amino acids Leu and Ser, it is not clear if N45 is located on the variable arm, or after it. Since our analysis shows a strong ability of other positions on the molecule to provide good models, we chose to leave out the variable arm from the analysis in the paper.

## 3. The association between nucleotides at pairs of tRNA positions

Cramér's V (or phi) (Cramér, 1946) was used to quantify the association between each pair of tRNA positions from the 43 positions. The results are visualized using a corrgram (Friendly, 2002) of a Cramér's V matrix over all of the combinations of the 43 positions (see Figure S6), produced using the R package *corrplot* (Wei, 2013).

A randomization test (with 100,000 repetitions) was used to estimate the p-values for table cells containing less than 20 counts. The order of the rows and columns of the positions in the displayed correlation matrix corresponds to its order on the tRNA molecule. The Bonferroni adjusted non-significant correlations at p ≤ 0.01 were not plotted.

The correlations between the Watson-Crick paired nucleotides in the three main stems (acceptor stem, TPC-stem, D-stem) have the highest correlations (inside the 3 squares), but the Watson-Crick bonds are the weakest in the D-stem, between positions N13-N22 and N10-N25. In general, positions on one part are not highly correlated with a positions on any other part (r ≤ 0.5), except for N47 on the right, which is correlated with the positions on the left.

*Fig. S6: A “corrgram” of pie charts displays Crammer’s V correlation matrix for measuring the association of nucleotides located at different positions on the tRNA (for 86 Archaea). The colors for the pie charts range from green-grey (no correlation), through yellow-orange (0.5 correlation) to red (full correlation). Non-significant correlations are not displayed.*

## 4. The use of the CART algorithm

The following shows the results of Figure 1 for more positions:

*Fig. S7: The prediction accuracy of the CART model that is based on sequentially adding tRNA nucleotides' positions*

## 5. Distances between species based on tRNA ensembles

While the use of distances between segments is very common in many standard tools, the current problem raises the need to construct a special distance to reflect the distance between any two species, as represented by their ensembles of tRNA-amino acid pairs. Some of the unique challenges here are: (i) Two identical segments ligating different amino acids in different species are very far apart; (ii) Different species might use a different number of tRNA molecules as moRNA code for the same amino acid, which also results in a variability in the total size of the tRNA system.

We first define the distance between two aligned moRNA determinants of same length in the simplest way, as the Hamming distance *h(x,y)*. Then, define the distance between one moRNA *x* and a set of moRNAs *S*, all encoding the same ligated amino acid by $d\left(x,S\right)=\min\left\{h\left(x,s\right)|s\in S\right\}$

(Note that *x* is contained in the set *S* if, and only if, $d\left(x,S\right)=0$.)

Next, for two sets of moRNAs *S_1_^aa^* and *S_2_^aa^,* both encoding the same amino acid *aa* in two species, we define the distance between them as the sum of the distances of each member of *S_1_* to the set *S_2_* plus the sum of the distances of each member of *S_2_* to the set *S_1_* :

.

Note that:

.

Finally, the distance between the ensemble $E_{1}=\cup S_{1}^{aa}$ of one Archaea and the ensemble $E_{2}=\cup S_{2}^{aa}$of a second Archaea is the mean distance, over all amino acids, between the corresponding sets: $d\left(E_{1},E_{2}\right)=\sum_{a=1}^{20} d\left(S_{1}^{aa},S_{2}^{aa}\right)$.

Combining all four stages into one, we can write the tRNA-ensemble distance as

$$\begin{matrix} d\left(E_{1},E_{2}\right)=\frac{\sum_{aa=1}^{N_{aa}} d\left(S_{1}^{aa},S_{2}^{aa}\right)}{N_{a}}=\frac{\sum_{aa=1}^{20} \left[\sum_{x\in S_{1}^{aa}} d\left(x,S_{2}^{aa}\right)+\sum_{y\in S_{2}^{a}} d\left(S_{1}^{aa},y\right)\right]}{20}= \\ \frac{\sum_{aa=1}^{20} \left[\sum_{x\in S_{1}^{aa}} \min\left\{h\left(x,s_{2}\right)|s_{2}\in S_{2}^{aa}\right\}+\sum_{y\in S_{2}^{aa}} \min\left\{h\left(y,s_{1}\right)|s_{1}\in S_{1}^{aa}\right\}\right]}{20} \end{matrix}$$

The distance thus defined enjoys several desirable properties:

(i) It is symmetric, i.e., $d\left(E_{1},E_{2}\right)=d\left(E_{2},E_{1}\right)$.

(ii) The distance between two ensembles is 0 if, and only if, the two ensembles are identical.

(iii) If $E_{1}\subset E_{2}\subset E_{3}$, then $d\left(E_{1},E_{2}\right)\leq d\left(E_{1},E_{3}\right)$.

(iv) The distance *d* may not fulfill the triangle inequality, for instance, in cases with a different number of sequences, or of having missing tRNA sequences for some of the amino acids in the compared ensembles. For the data at hand, the above-defined semi-distance was empirically found to be a distance over all triplets.

## 6. Constructing a phylogenic trees

This is detailed in the methods section of the paper.

## 7. Measuring the similarity of two phylogenetic trees (Baker's gamma) – per AA

| Sequences used for  constructing the  moRNA phylogenetic tree | Subset of sequences  only encoding for | Number of unique  species discerned  by the moRNA phylogenetic tree | Baker’s Gamma rho |
| --- | --- | --- | --- |
| Best 10 position | All amino acids | 72 | 0.856 |
| Best 8 position | All amino acids | 72 | 0.727 |
| Best 6 position | All amino acids | 72 | 0.625 |
| Best 3 position | All amino acids | 65 | 0.657 |
| Best 8 position | Val | 20 | 0.708 |
| Best 8 position | Tyr | 12 | 0.098 |
| Best 8 position | Trp | 16 | 0.350 |
| Best 8 position | Thr | 29 | -0.097 |
| Best 8 position | Ser | 46 | 0.083 |
| Best 8 position | Pro | 23 | 0.826 |
| Best 8 position | Phe | 5 | 0.153 |
| Best 8 position | Met | 30 | 0.513 |
| Best 8 position | Lys | 13 | 0.170 |
| Best 8 position | Leu | 36 | 0.059 |
| Best 8 position | Ile | 11 | 0.119 |
| Best 8 position | His | 21 | 0.611 |
| Best 8 position | Gly | 34 | 0.173 |
| Best 8 position | Glu | 19 | 0.660 |
| Best 8 position | Gln | 26 | 0.468 |
| Best 8 position | Cys | 23 | 0.300 |
| Best 8 position | Asp | 13 | 0.792 |
| Best 8 position | Asn | 5 | 0.321 |
| Best 8 position | Arg | 53 | 0.353 |
| Best 8 position | Ala | 25 | 0.528 |

Table. S1: Baker's gamma for the association between the out-of-tRNA tree and the moRNA trees, constructed in different ways: with a different number of best position used (sequence length) and number of sequences used for the tree.

## 8. (K, Bk) plots –definitions and illustrations

For a closer look at the comparison of the moRNA-ensembles tree and outside-tRNA tree one can resort to the FM (or Bk) method (Fowlkes & Mallows, 1983) in which for each *k* (k=2,3,…,73) the association between the two trees, when trimmed to *k* leaves, is evaluated (see Figure S10).

We further explore the similarity between two hierarchical clustering dendrograms using the *(k,Bk)* plot, suggested by Fowlkes and Mallows. For every level of splitting of the two dendrograms which produces *k* clusters in each tree, the plot shows the number *Bk*, and therefore enables the investigation of potential nuances in the structure of similarity. The *Bk* measures the number of pairs of items which are in the same cluster in both dendrograms, one of the clusters in one of the trees and one of the clusters in the other tree, divided by the geometric mean of the number of pairs of items which are in the same cluster in each tree. Namely, *a_uv_=1* (*b_uv_=1*) if the items u and v are in the same cluster in the first tree (second tree), when it is cut so to give *k* clusters, and otherwise *0*:

The *Bk* measure can be plotted for every value of *k* (except *k=n*) in order to create the “(*k,Bk*) plot”. The plot compares the similarity of the two trees for different cuts. The mean and variance of *Bk*, under the null hypothesis (that the two trees are not “similar”), and under the assumption that the margins of the matching matrix are fixed, are given in Fowlkes and Mallows. They allow making inference on whether the results obtained are different from what would have been expected under the null hypothesis.

The plot of *Bk* for all *k*'s, which further displays the expected *Bk* and the 95% confidence region under the ‘no association’ assumption, is given in SI 8. Three peaks are evident in this plot, at *k*=3, 8, and 32, with Bk=0.859, 0.763, and 0.582, respectively. The high value of *Bk* at these cuts may reflect different aspects. For *k=3*, the moRNA-ensemble phylogeny tree is able to detect that the species in our dataset are divided into three major families (Crenarchaeota, Euryarchaeota, and other). For k=8, the tree gives a good separation into about 8 sub-families of species: 7 very frequent beginning of names which are also neighbors in the standard phylogenetic tree (these are: Thermococcus, Pyrococcus, Methano…, Halo...., Methano..., Pyrobaculum..., Sulfolobus). So it seems that the moRNA-phylogeny tree reflects wider properties of the species evolutionary origin. For k=32, each leaf includes 2 species on the average; the high level of *Bk* may reflect that the moRNA-ensemble distance is able to detect, fairly well, pairs of species that are evolutionarily close.

The evolution of the moRNA determinant ensembles per individual amino acids can also be studied in a similar way. The phylogenetic trees thus generated vary in their correlation with the out-of-the-tRNA tree from practically none (for Thr, Leu, Ser, Tyr) all the way up to 0.82 for Pro, with a median of 0.335 (see Table S1). However, for each amino acid ensemble separately, the partition of the species is much courser than that of the combined ensemble, and therefore far from unique. Consequently, these correlations are not directly comparable. Trying to explain the different levels of similarity for different amino acids by the number of species sets they identify, or by the number of nucleotides of high importance involved in their identification, revealed no clear pattern. One can trace the effect of the first partition into families on the determinants of each amino acid using Figure S9-S11.

*Fig. S8: (k, Bk) plot, comparing the phylogenetic tree based on information outside the tRNA and the tree based on the 8-nucleotides-long moRNA cipher. The Bk line (circled), and the expected Bk (in bold) and 95% confidence region (dashed) under the ‘no association’ assumption. The Bk line is significantly above the upper dashed line.*

## 9. Visualizing the moRNA cipher

There is no simple way to clearly visualize the complex moRNA cipher for a specific set of species. One approach is to create a panel of sequence logo plots, one for each of the ligated amino acids. Next, we present these plots for all species, and two other plots for each of the two main families of Archaea.

While some patterns are easy to detect (for example, how only Ala uses "T" in position N70), it is important to note that interactions between the position will not be visible in these plots.


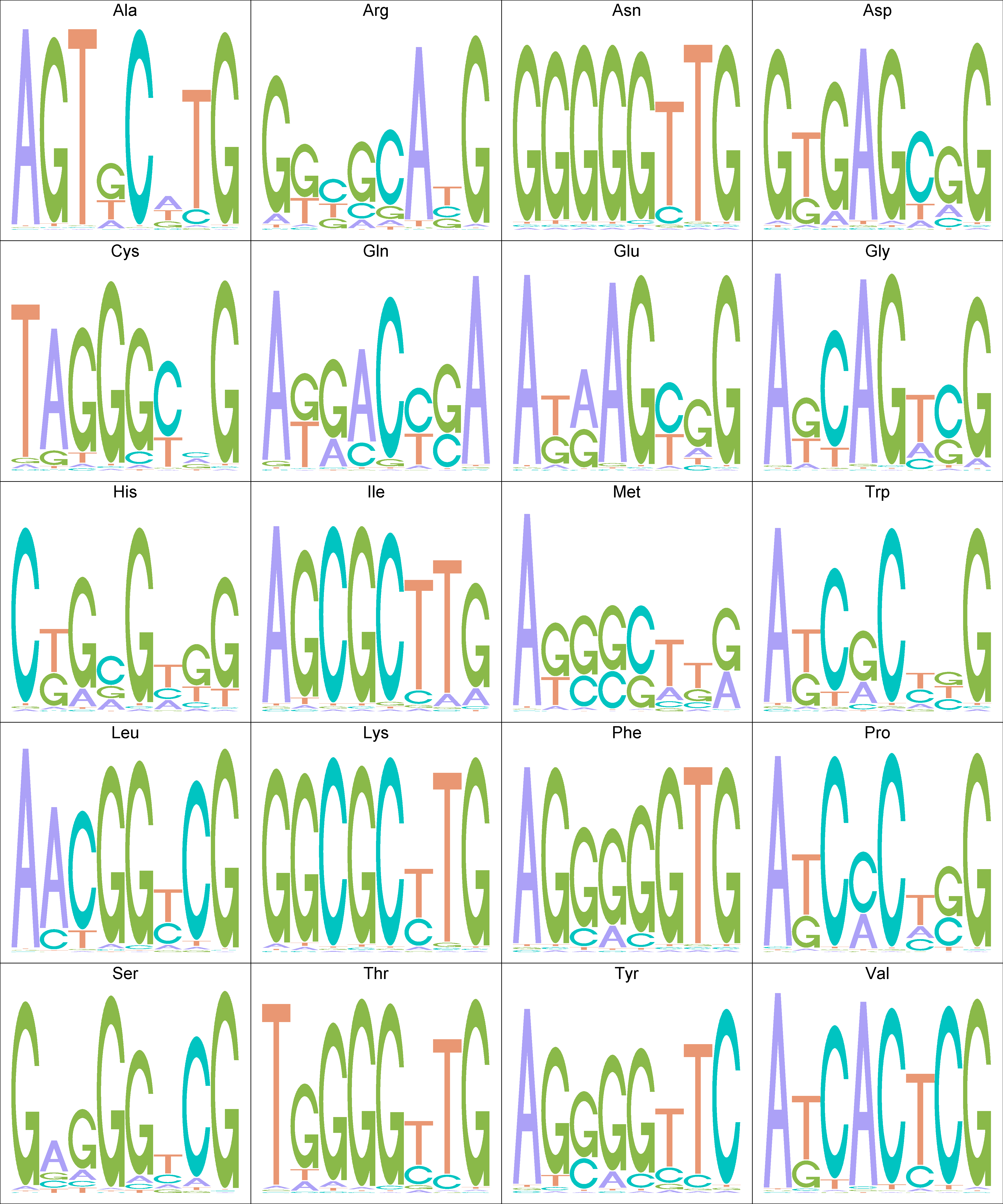


*Fig. S9: Facet sequence logo plot for 86 Archaea species based on positions (from left to right) "N73, N22, N70, N24, N71, N20, N12, N1"*


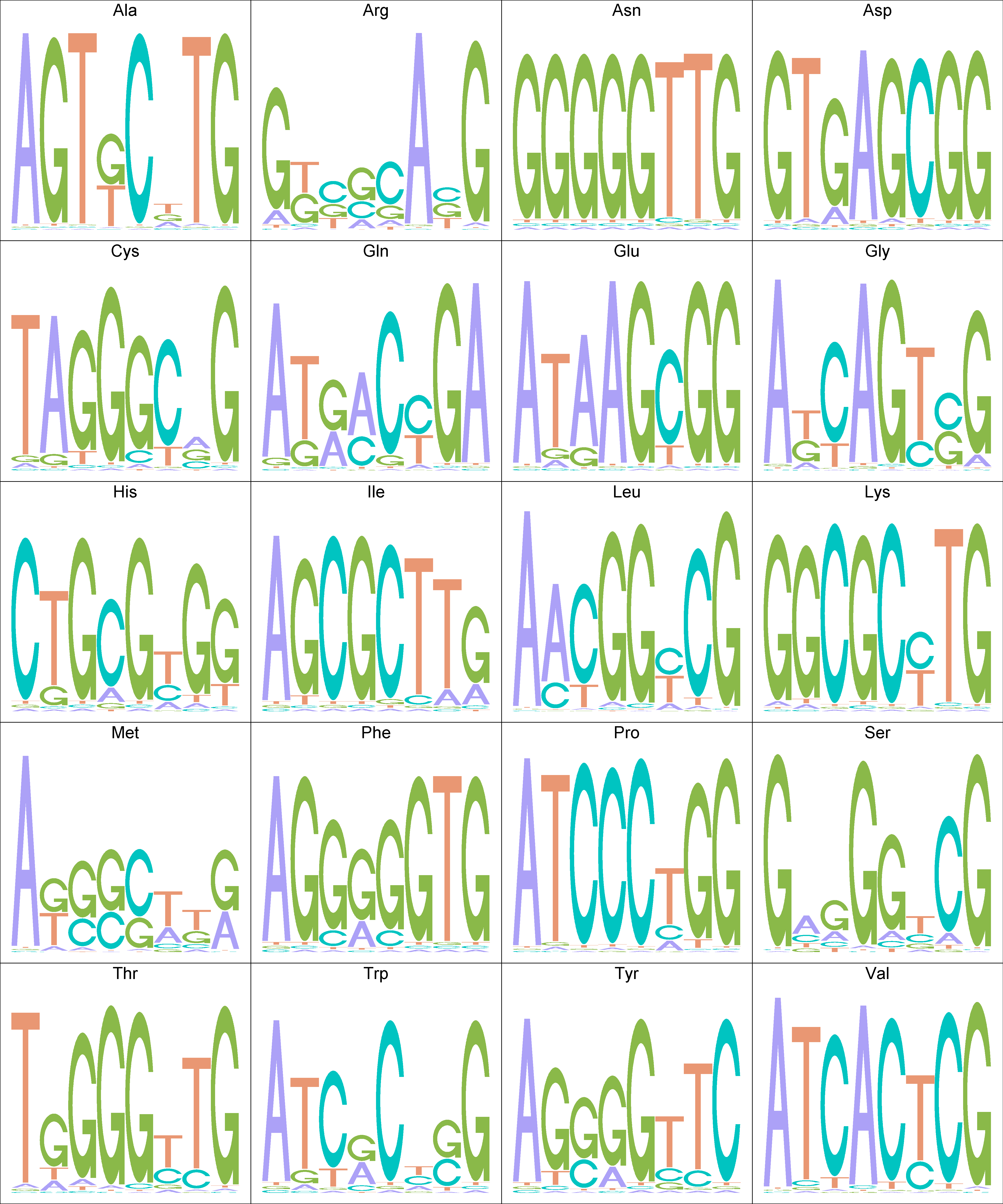


*Fig. S10: Facet sequence logo plot for 57 species (2597 tRNA) from the Euryarchaeota family, based on positions (from left to right) "N73, N22, N70, N24, N71, N20, N12, N1"*


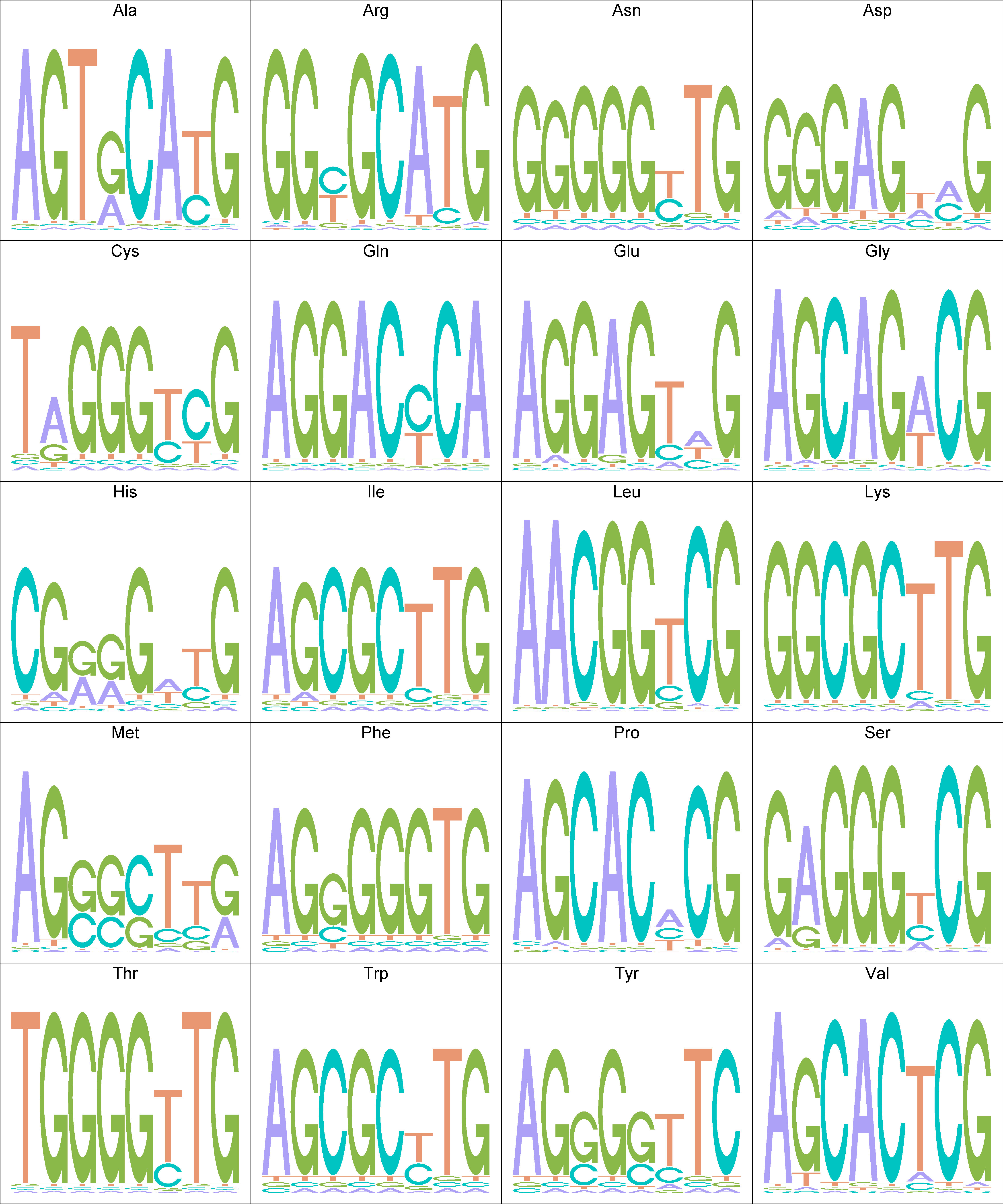


*Fig. S11: Facet sequence logo plot for 25 species (1159 tRNA) from the Crenarchaeota family, based on positions (from left to right) "N73, N22, N70, N24, N71, N20, N12, N1"*

# 10. References

Bembom, O. (n.d.). seqLogo: Sequence logos for DNA sequence alignments.

Ciliberto, G., Raugei, G., Costanzo, F., Dente, L., & Cortese, R. (1983). Common and interchangeable elements in the promoters of genes transcribed by RNA polymerase III. *Cell*, *32*(3), 725–733.

Cramér, H. (1946). Mathematical Methods of Statistics (Princeton. *Press, Princeton, NJ*, 282.

Fowlkes, E. B., & Mallows, C. L. (1983). A Method for Comparing Two Hierarchical Clusterings. *Journal of the American Statistical Association*, *78*(383), 553 – 569. Retrieved from http://www.jstor.org/stable/2288117

Friendly, M. (2002). Corrgrams. *The American Statistician*, *56*(4), 316–324. doi:10.1198/000313002533

Marck, C., Kachouri-Lafond, R., Lafontaine, I., Westhof, E., Dujon, B., & Grosjean, H. (2006). The RNA polymerase III-dependent family of genes in hemiascomycetes: comparative RNomics, decoding strategies, transcription and evolutionary implications. *Nucleic Acids Research*, *34*(6), 1816–1835.

Wei, T. (2013). corrplot: Visualization of a correlation matrix. Retrieved from http://cran.r-project.org/package=corrplot
